# A Treatise on Indigestion and Its Consequences, Called Bilious and Nervous Complaints, &c.

**Published:** 1821-09-01

**Authors:** 


*A Treatise on Indigestion, and its Consequences, called Nervous and Bilious Complaints; with Observations on the Organic Diseases, in which they sometimes terminate.* By A. P. W. Philip, M. D. F. R. S.. Ed. &c. One vol. 8vo, pp. 363. London, 1821.

The number of works which have lately issued from the press, under titles nearly similar to the above, sufficiently prove that the subject is exciting more attention than formerly. On the Continent, as our readers well know, almost all fevers, and most of the acute eruptive diseases, are supposed to have their sources in the primæ viæ; while, in this country, almost every chronic complaint, as well as many of the phlegmasiæ, are traced, or thought to be traced, to the digestive organs. Although the most rational views and the soundest doctrines are sure to be perverted and misapplied by the less reflecting and less discriminating part of the profession—(and that must always be a very large proportion)—yet we are very far from approving that satirical levity, bordering on caricature, with which some people treat this highly important and interesting subject. Charlatanism and knavery deserve no quarter, and require the rod of ridicule; but well meaning efforts at improvement, and undisguised opinions, however erroneous, are not, we apprehend, fit subjects for licentious criticism, or ill-timed wit.

If we seriously reflect that there are but two surfaces, the external and internal—or, in other words, the dermoid and mucous expansions, on which all physical agents must make their first impressions, and through which they must pass in their way to all or any other parts of the system, we shall find but little cause to laugh at the supposed extravagance of those who attribute the origin of almost all morbid affections to changes, whether of function or structure, in the two surfaces abovementioned. Look at the great tribe of acute diseases, whose etiology and phenomena are more evident than those of the chronic, and you will perceive *disturbances of function* in one or both of the surfaces in question, among the first, if not the very first, effects of the external morbid cause. Whether these causes radiate from the said surfaces to other parts of the system through the medium of nerves, blood-vessels, absorbents, or all three, may admit of dispute; but no one can deny the fact that they *do* radiate thence. Even in those diseases originating in moral or mental emotions, the functions of the mucous membranes and skin are among the very first to be deranged. In all points of view, then, the study of functional and organic lesions of the digestive apparatus is by far the most important to which the physician can apply himself. Unless we trace diseases through their earlier as well as ulterior steps, we shall be constantly travelling in the dark, and stumbling from one error to another.

In our last number, we gave a comprehensive view of those graver affections of the mucous membranes of the digestive organs, which come under the designation of Phlogosis and high irritation; but there is a wide range of functional affections of the stomach and associated organs, short, in most instances, of decided inflammation, yet consisting of irritation or deranged action that not seldom rises to or ends in phlogosis and change of structure, either in the viscera primarily concerned, or in those which are drawn into disorder through sympathetic association with them. These are treated of in the work before us under one comprehensive generic term—INDIGESTION, which Dr. Philip uses, not as synonymous with dyspepsia, but including it, and also what are termed bilious, nervous, and stomach complaints. From this annunciation our readers will readily perceive the extent of the subject embraced in the volume under review, and be prepared to expect an analytical article of corresponding dimensions.

The work naturally divides itself into three parts, namely, the symptomatology, etiology, and treatment of indigestion; to which is added a fourth part, or chapter, on dyspeptic phthisis, and habitual asthma.

I. *Symptomatology*. It has long been observed, that the symptoms of indigestion are numerous and often anomalous, beyond all hope or power of description. We shall not therefore, in this analysis, advert to more than the prominent and less mutable traits of this proteiform malady. In the incipient stage of indigestion, the symptoms are such as seem to arise from the irritation of the food imperfectly acted on by the stomach, and of its own vitiated secretions. Those of the first class are chiefly flatulence, distension, eructations of oily, rancid, or putrescent character. These may continue a considerable time, without extending their influence to other parts of the system, or, in fact, preventing the enjoyment of otherwise good health. These symptoms may often be prevented or removed by a more restricted and regulated diet than before. When they are neglected, however, or when they are of a very obstinate character, the secretions of the stomach and bowels begin to suffer deterioration. The bowels do not act so readily as usual—are occasionally distended and tense—with clamminess of the mouth, and more or less whiteness of the tongue in the mornings. Even these symptoms will generally yield to some mild aperient or alterative medicine. But by degrees they recur more frequently, and begin to be attended with some depression of strength, which is usually the first thing which excites the attention of the patient. If the disease proceed, the mind partakes of these languors, and the patient finds that he is not equal to his usual mental efforts. He feels occasional despondency, and becomes more alarmed than necessary respecting his complaint.

"While the symptoms thus proceed, a change, sooner or later, takes place, which marks an important step in the progress of the malady. The alvine discharge begins to deviate from the healthy appearance: it sometimes contains uncombined bile, sometimes it chiefly consists of bile; its colour at other times is too light, more frequently too dark, and occasionally, at length, almost black; at different times it assumes various hues, sometimes inclining to green, sometimes to blue, and sometimes it is mixed with, and now and then almost wholly consists of, undigested bits of food. It often separates from the canal with more difficulty than usual, and leaves a feeling of the bowels not having been completely emptied. These symptoms only mark a greater degree of what has been going on from the first." 12.

Our author believes, with reason, that the above change and variety of colour arise chiefly from the state of the bile —and "that if the colour of the alvine discharge be natural, we may generally infer that the function of the liver is duly performed." Certain kinds of diet, and even the remora of bile in the bowels will occasionally alter the colour of the alvine discharges:—thus a milk diet produces lighter coloured fæces than one of animal food; yet the sechanges are but trifling, and hardly to be taken into the account. In indigestion the urine often throws down lateritious or white sediments, and sometimes exhibits on its surface a thin oily film, which may arise from imperfect assimilation of the food. In states of nervous excitement every one knows that the urine is limped and in very large quantities.

"A remarkable sympathy between the state of the kidneys and intestines is often observed in indigestion, the urine remaining scanty and high-coloured, when the bowels are constipated; and flowing freely, and of a pale colour, as soon as a free discharge from them has been obtained. Even in those dropsical affections which supervene on this disease, it is common for all diuretics to fail, when the bowels are constipated, and for the operation of cathartics alone to be followed by a free discharge from the kidneys." 16.

The vicarious action of the skin, kidneys, and intestines, is well known. When the skin has become torpid in delicate children, the bowels will often discharge astonishing quantities, solid as well as fluid—and that even, when little nourishment is taken in. In such cases, our author has reason to think the inhalation by the skin to be inordinately great. He saw several gallons of water drawn off from a child ten or twelve years old, labouring under abdominal disease, re-collected in eight or ten days, although but little fluid had been drunk. The opposite state of the system is well illustrated by the cases of many gluttons whose alvine excretions are not greater than those of temperate eaters, while the cutaneous transpiration is abundant.

"The sensible change in the appearance of the alvine secretions in indigestion, is generally attended with some change in the other symptoms. The bowels are more frequently variable, diarrhœa often supervening without any evident cause, almost uniformly followed by fits of constipation. These, the patient finds, cannot now be removed by the simple medicines which at first restored due action to the bowels, larger doses or more active medicines are necessary, and their effect corresponds with the previous state of the bowels. The discharge is generally unsatisfactory, something seeming to be retained. It is very often watery, or frequent small semi-fluid, teasing, mixed with mucus, and sometimes streaked with blood, and after it has been repeated, often chiefly consists of mucus and a little blood, the passage of which is attended with much griping and bearing down, and followed by a constant desire of further evacuation. The patient takes more medicine, with the hopes of a freer effect, but he thus often increases the straining more than the discharge." 19.

The morbid contents of the alimentary canal now produce pains in the stomach and bowels, weight in the right hypochondrium, with distension there, or in the lower part of the abdomen, foul and clammy tongue, nausea, depression of strength, and remarkable despondency of mind.

The play of the morbid sympathies next becomes manifest, by affections of the head, the senses, or the muscles. These prey on the patient's mind, which, reacting on the body, still farther debilitates the digestive organs, and aggravates the other symptoms. The chylopoetic viscera being no longer in a proper condition to supply due nourishment, emaciation necessarily succeeds, while the countenance, a true index of what is passing internally, becomes pale and haggard. Often at an early period there is decubitus difficilis of the *left*, more rarely of the *right* side. In the progress of the disease, the *decubitus facilis* is on the back with the shoulders a little raised. It is reasonable to expect that in derangements of the stomach and digestive viscera, those parts of the system most connected with them in function, will be those most severely catenated in disease. Thus the tongue and other parts of the mouth are variously affected from the commencement. Their secretions become thick and clammy, or deficient, or morbidly thin and copious.

"In some cases the tongue, in the more advanced stages, becomes clean, shining, and morbidly smooth, and at length affected with aphthæ. This state of it is seldom observed except when a considerable degree of fever has supervened, which is not uncommon at these periods." 25.

The skin, in protracted cases, often becomes dry and shrivelled, the surface being cold, the patient chilly, and bearing ill all atmospheric vicissitudes. The head often indicates undue determination of blood to that quarter, producing languid ophthalmia, tinnitus aurium, and sometimes throbbing of the temples, drowsiness, cephalalgia, giddiness, or stupor. The thoracic viscera are often particularly affected, as might be expected from contiguity of position, association of function, and connexion of nerves. Among the principal sympathetic phenomena are, dyspnœa more or less permanent, dry irritating cough, palpitation, pains in various parts of the chest. At different periods of the disease, and after repeated derangements of the hepatic function, permanent tenderness on pressure is complained of about the cartilages of the right false ribs.

"This symptom never exists long and to any considerable degree without the pulse becoming hard, and it often at the same time becomes rather more frequent than in health. There is no other symptom of the disease before us to which I am so anxious to call the reader's attention as to what I have here termed a hard pulse, because on it much of the proper treatment seems to depend. It sometimes happens, especially when the tenderness in the epigastrium is considerable, that the pulse becomes such as would on all occasions obtain the name of hard; but more frequently the hardness is only to be distinctly perceived by examining the pulse in a particular way.

"Those who have been much in the habit of examining the different states of the pulse must be aware, that its hardness is most perceptible when a slight degree of pressure is employed. A certain degree, by greatly compressing the vessel, will give some feeling of softness to the hardest pulse, and a slight degree of hardness is not perceptible with the pressure generally employed in feeling the pulse. If the pressure be gradually lessened till it comes to nothing, it often happens that a distinct hardness of pulse will be felt before the pulse wholly vanishes under the finger, when no hardness can be distinguished in the usual way of feeling it.

"This is in no degree the case in a healthy pulse, nor even in the first stage of the disease we are considering. But when the tenderness of the epigastrium is at all a prominent feature, it may always be perceived, that is, there is then a certain degree of pressure, sometimes very slight, under which the pulse gives a decidedly wiry sensation to the finger, the degree of pressure, under which the hardness may be perceived, denoting its degree." 29.

This concurrence of epigastric tenderness and hard pulse our author considers as the commencement of a new or second stage, wherein the nature and treatment of the disease are changed. The epigastric tenderness may readily be recognized, but the minute distinction of pulse, we fear, is but little calculated for the general *run* of practice, however useful for those who attend minutely to every phenomenon connected with disease.

With the two symptoms abovementioned, there are generally others ushered in, indicative of febrile movements in the system. The chilliness long complained of is now sometimes interrupted by languid and oppressive flushes of heat—the hand and feet often burning, particularly in the early part of the night—with increase of thirst, and sometimes partial morning sweats.

"When the tenderness of the epigastrium and hard pulse are considerable, there is generally more or less an inability of exercise, except of the passive kind, much active exercise producing an insupportable languor. Slight degrees of the above symptoms are generally unattended by this inability." 30.

Epigastric tenderness generally ends in epigastric and hypochondriac fulness, and even some degree of firmness, with pain passing in various directions on pressure.

Such is the regular course of what our author has observed in the first and second stages of indigestion, independently of the remote or sympathetic affections of distant parts, which too generally spring up during the progress of these two stages. In the first stage of indigestion, Dr. Philip considers these as purely nervous, disappearing as soon as their causes cease to operate. In the second stage they become more and more permanent; "and in the same proportion, more independent of the original disease, till at length they cannot be removed without an appropriate mode of treatment directed to the part secondarily affected."

In all these affections, as our author justly observes, both the sanguiferous and nervous functions are involved, and this cannot continue long without change of structure. They are therefore the immediate forerunners of organic disease, and if they cannot be arrested, the last stage of indigestion is established.

"For it is a curious fact, and one of the greatest importance in the treatment, that the organic affection rarely takes place in the original seat of the disease, but in other organs with which the stomach sympathizes, the liver, pancreas, spleen, mesenteric glands, lower bowels, heart, lungs, brain," &c. 34.

Thus, when the body is examined after death, the patient is said to have died of disease of some of the abovementioned parts, and there is nothing in the appearance of the organs in such cases to distinguish them from diseases which originate in the organs themselves. It is by the history alone that we discriminate the sympathetic from the idiopathic disease.

"It is one of the curious circumstances of the progress of the disease we are considering, which particularly demands attention, that when it fixes decidedly on one organ, the others are, to a certain degree, and sometimes wholly, relieved. The establishment of all the above secondary affections generally relieves the dyspeptic symptoms; and, even a secondary disease may be relieved by another supervening on it.

"Thus, it is not uncommon in indigestion for the liver to suffer in such a manner, that it shall become enlarged and tender on pressure, and when the disease is destroying the texture of the lungs, having spread from the liver to them, for the former to recover, or nearly recover its healthy state. Symptomatic disease, when completely established, seems to act on that which excites it, in the same way, though much more effectually, in which artificial drains are found to do, while the sympathetic affection which precedes the establishment of actual disease, tends to increase the original derangement. Thus also an extensive external disease, as I have witnessed, occurring in such cases, will often save the vital organ even after the disease has made considerable progress in it." 36.

We are no more able to explain why, by artificially excited external disease, we can so feebly imitate the effects of a spontaneous occurrence of it, than why a spontaneous sweat carries off fever, while one produced by art often brings little, or but partial relief.

Indigestion, then, according to our author, may be divided into three stages:—the *first*, characterized by the various symptoms described, as arising from undigested food, and the irritation of vitiated secretions:—the *second*, characterized by tenderness in epigastrio, and a degree of hardness in the pulse, often accompanied by febrile symptoms:—the *last*, by symptoms of organic disease in the head, chest, or abdomen.

We have some doubts, in our own minds, whether Dr. Philip be *pathologically* correct in separating the second and third stages. To us they appear the same in kind—that is, we conceive that the structure begins to suffer from the commencement of the second stage, and consequently that there are, pathologically, but two stages, the functional and organic; each stage having its various degrees of advancement, according to the length of time it has continued. In a therapeutical point of view, however, we have no objection to Dr. Philip's arrangement.

The relative duration of these different stages of indigestion, as well as the severity and nature of their symptoms, is very various. In its ulterior stage, too, it assumes a great variety of forms, according to the organ or texture organically and sympathetically affected.

"And it tends still further to perplex the symptoms, that in some cases the disease proceeds in more than one organ at the same time, the affection of the one not arresting that of the other, as we have seen, sometimes happens." 44.

In every stage, in short, there is endless variety, and the nearer it approaches the fatal termination, "the more its different cases assume the appearance of diseases which have nothing in common." The reason why one part should be sympathetically affected, in preference to another, is probably because it is weaker, and more predisposed to disease in general—at least we can form no other conjecture.

When gout is excited, as symptomatic of mal-function of the digestive organs, Dr. Philip considers it as tending less to derange the system in general, and more productive of relief to the primary disease, than most of the other symptomatic affections.

> "Hence appears the danger which attends interrupting the regular fits of gout, the sympathetic disease being prevented from taking the course which the disposition to affection of the extremities gives it, seizes on the part, generally an internal one, which next to these is most liable to disease; and on the other hand, if any thing so affects any of the vital parts during a fit of gout as to render it considerably the weakest part, the sympathetic disease sometime leaves the joints and seizes on the internal part, producing what is called retrocedent gout. It is evident that the risk of both these accidents will be greatest, where the powers of the system are most impaired." 47.

In respect to the connexion of indigestion and urinary gravel, we exhibited a full account of Dr. Philip's observations and experiments at page 623, *et seq*. of the first volume of this Journal, while reviewing the 6th volume of the College Transactions; we need not therefore repeat them here. As to the third stage of indigestion—namely, that in which it has produced organic affections, it is foreign to our author's purpose to enter into its minute consideration, as it would lead to an investigation of a "large proportion of all the most serious diseases to which we are subject." Towards the close of the work, however, this topic will be taken up, as far as consistent with the plan of the present publication.

II. *Etiology*. In entering on this part of the subject, Dr. Philip introduces many ingenious physiological observations on digestion, which we cannot abridge without injuring them, and therefore must refer to the work itself. The remote causes of indigestion are properly divided by our author, into those which act directly on the stomach and intestines, those which act on other parts, and those which affect the whole system. He justly remarks that, as the affections of parts, which sympathize primarily or secondarily, influence the state of each other, so the sympathetic affections produced by disease become causes which support and aggravate it. Thus the debility of the skin, occasioned by indigestion, so reacts on the digestive organs as to increase the disease of the stomach; and similar observations apply to affections of the liver, brain, and other parts produced by gastric disorder. The evil thus increasing in a kind of geometrical ratio the whole powers of the system, in severe attacks, often sink with a rapidity which, at first view, appears unaccountable. It appears to our author that the function of the stomach may be deranged by causes which either affect its secreting power, so that the proper chemical changes are no longer effected, or debilitate its muscular power, so that the food, though digested, is not regularly propelled into the duodenum. Among the chief causes of indigestion acting directly on the gastric muscular fibre, may be enumerated narcotic and other offensive substances received into the stomach, as opium, tobacco, distilled spirits, or matters of an acid or putrid nature generated in the organ itself. *Morbid distention* of the stomach is regarded by Dr. Philip as one of the most frequent and powerful causes of indigestion—and *that* is generally produced by eating too fast:—

"For the appetite only subsiding in proportion as the food combines with and neutralizes the gastric fluid, previously in the stomach, when we eat too fast, time is not given for it to combine with that part of the food which is presented to it, till so much is taken that the whole gastric fluid, which the stomach is capable of supplying during the digestive process, is not sufficient to effect the due alteration on it; whereas, when we eat slowly, so that a proper time is given for the combination to take place, the appetite abates before the stomach is overcharged; for while digestion goes on, and the gastric fluid is only supplied in proportion as fresh food comes in contact with the coats of the stomach, it combines with the food as it is formed, and never excites the appetite." 76.

Every one knows that an interruption of ten or fifteen minutes, in the middle of a meal, will sometimes remove the desire for more food; and it is for the same reason that a few mouthfuls taken a little before dinner, will often wholly destroy the appetite, especially in delicate people, in whom the gastric fluid appears to be secreted in small quantity, or of inactive quality. Another frequent cause of over-distention is high seasoning and great variety of food; which either induce us to eat too long, or excite a greater supply of gastric fluid than the food calls for, by which the appetite is prolonged. The latter is exemplified in the effects of wine drunk during dinner. The degree of distention, too, depends much on the *kind* of aliment. All food swells more or less, from the heat and moisture; but that which is most difficult of digestion swells most. Over-distension of the stomach, however, must prove injurious to the nerves of that organ, and thus produce that peculiar pain, restlessness, and sense of oppression, which attend an over-distended stomach. "Such irritation of the nerves of a secreting surface cannot exist without affecting its secreting power." The gastric fluid therefore becomes less fitted for its functions, and thus the distention is increased. There are many causes, however, which derange the nervous power of the stomach, and thus vitiate its secretions, by impressions made wholly on the nervous system, and only secondarily on the muscular fibres of the stomach. Among these are reckoned violent passions of the mind, too close application to study or business, excessive venereal indulgences.

"Strong impressions of the mind often instantly destroy the appetite, by occasioning such a secretion of gastric fluid as, not possessing healthy properties, at once itself fails to apply the due stimulus to the stomach, and tends to vitiate the effect of that which had been previously secreted." 80.

In intoxication the stomach would appear to suffer not only from the over-excitement of the inebriating fluid, but in consequence of the cerebral disturbance produced by the same cause.

"Similar observations apply to a moist, cold, and variable atmosphere. The stomach not only suffers by the general debility and relaxation induced on the nervous, and, through it, on the muscular system; but also by the peculiar effects of such an atmosphere on the office of the skin. Thus, too free a use of calomel and other medicines which powerfully affect the abdominal secretions, not only injures the stomach by their direct effect on this organ, but by the disorder excited in parts with which it immediately sympathizes. So extensive, indeed, are the sympathies of the stomach, that whatever greatly disorders the function of any important organ, may be ranked among the causes of indigestion; its tendency to produce this disease being proportioned to its degree, and the degree of sympathy which exists between the stomach and the part primarily affected." 82.

Affections of the bowels, bladder, urethra, head, or other parts, will sympathetically produce derangement of the stomach. There can be little doubt also, that *hereditary* predisposition to indigestion has its weight in the scale of causation.*

The *immediate* causes of a disease, according to Dr. Philip, are the states of body induced by the remote causes, and from which all the symptoms, more or less, directly arise.

---

* "All the viscera," says M. Merat, "are linked together by a general sympathy, (consensus general,) and therefore affections of the brain, lungs, womb, bladder, &c. act on the digestive organs, and interrupt the process of digestion. The *skin* also reacts strongly on the stomach."—*Dict. des Sciences Med. Tom.* 24.

This, in fact, is the disease itself. It is evident, our author thinks, that the causes of indigestion must necessarily affect either the muscular or nervous power of the stomach, or both, on which its functions depend.

"As the food can only be regularly propelled into the duodenum by the due action of the muscular power of the stomach, it is evident that, if this fails, oppression and distension must ensue; and if the due secretion of the gastric fluid depends on the healthy state of the nervous influence of the stomach, its properties must be affected by any cause disordering this influence, and the food, consequently, will not be duly changed by it." 88.

Dr. Philip is of the same opinion with some of our ablest physiologists, as mentioned several times in this Journal, that nervous sympathy is produced, not by the communication of nerves *in their course*, but through the medium of the sensorium.

"These and various similar facts, as far as I can judge, leave no room to doubt, that nerves sympathize only from their connexion in their common source, and that the numerous connexions we observe in their course are only useful in the same way with the ganglions and plexuses, which may be proved by direct experiment, to enable the influence, descending from that source, to pass from one nerve to another, so that one may partake of that which is conveyed by many, a power, which it may also be shewn, is necessary to the continuance of life. That the phenomena of sympathy depend on changes in the source of nervous influence, might be proved by the fact alone, that sympathetic feelings still continue to be referred to a limb which is lost." 93.*

When the causes of gastric disease are comparatively slight, their effects on those parts which strongly sympathize with the stomach, may yet be very considerable, while on others they are hardly to be observed. "Thus, the effects of irritation of the stomach appear in the liver, the skin, the head, when they are not to be perceived in other parts; but as the disease increases in violence, they become sensible in every part." These observations are confirmed by the long known success which attends the treatment of many cutaneous diseases, by remedies and regimen adapted to the improvement of the digestive organs.

---

* Mr. Charles Bell very aptly illustrates this doctrine in his lectures, and those who carefully observe the phenomena of morbid sympathies, will be convinced of its truth. "I may here state it as my belief," says a recent writer, "that all sympathies are produced primarily through the medium of the nervous system—not from *connexion* of nerves, but through the brain." *On Derangements of the Liver, &c. p. 9.*

Dr. Philip endeavours to elucidate the pathology of the second stage of indigestion—namely, when epigastric tenderness and the hardness of pulse, before alluded to, have manifested themselves. These symptoms, he thinks, "indicate inflammation or a state approaching to it." He considers it therefore a point of considerable importance, in the treatment of this disease, to ascertain the nature and seat of the epigastric tenderness.

"It is evident that of the different parts of the stomach the pylorus is the one most exposed to the causes of irritation. Other parts experience the irritating effects of different portions of its morbid contents, but the pylorus is necessarily exposed to those of all. All must pass by this orifice. When therefore we see that, after indigestion has continued for some time, a certain part of the region of the stomach becomes tender on pressure, we cannot help turning our attention to this part. Now in the natural situation of the viscera, exactly in the tender part of the epigastrium, the pylorus lies, with the thin edge of the liver upon and in contact with it; as I have ascertained with the kind assistance of Mr. Brookes, whose anatomical skill is so generally acknowledged.

"When all these circumstances are considered, can we doubt that it is the irritated pylorus assuming a low degree of inflammatory action which occasions the tenderness on pressure above described; and when we consider what has just been said of the influence of juxtaposition in the spreading of this disease, can we doubt that when we find the tenderness with some degree of fulness gradually extending downwards, along the soft parts on the edge of the cartilages of the right side of the epigastrium, as we find it to do in the progress of indigestion, and at length ending in evident enlargement and tenderness of the liver, that the affection of the pylorus is communicated to the thin edge of this organ, with which it is in contact, thence by degrees extending to its other parts? Thus it is that of all the neighbouring parts no other so frequently partakes of this affection of the stomach. It is, doubtless, in the same way, that the habit of drinking spirits, which must apply so great a degreee of irritation to the pylorus, seldom fails to produce affections of the liver." 105.

We do not see any thing unfeasible in the above pathological reasoning, either in a theoretical or practical point of view. Our author thinks there is reason to believe that the pylorus itself is more subject to organic disease than any other part of the stomach, inasmuch as it is more exposed to causes of irritation; yet that it has a greater power of resisting disease, in consequence of which, the diseases propagated from the pylorus to the liver, "more readily take root there, (in the liver,) and the structure of this organ yields to the affection which that of the part, from which it receives it, generally resists."

"The liver indeed is here already disposed to disease, its action we have seen from an earlier period, having by sympathy been influenced by the state of the stomach, and the continuance of diseased action, we know, disposes to disease of structure." 106.

Dr. Philip believes, with Dr. Yeats, that fulness and tenderness of the right hypochondrium do not always indicate liver affection, but that, on the contrary, they sometimes depend on the state of the duodenum.*

"The feeling given to the hand by the distended duodenum is different from that produced by the gorged liver, and in the former case the chief fulness is generally lower down, and does not seem to proceed so immediately from under the edge of the thorax as general fulness of the liver does; so that, even where, from more permanent debility of the duodenum, its morbid distension is a more constant symptom, the two cases may generally be distinguished." 107.

Upon the whole, then, our author refers the *epigastric* tenderness to an inflammatory affection of the pylorus, excited by the passage of the irritating contents of the stomach, for a longer or shorter time—a tenderness which is generally confined, at first, to a space not larger that a shilling.

III. *Therapeia.* The great utility of etiology is its application to therapeutics; for the mainspring of curing a disease consists in the subduction of its causes. In the *first* stage of indigestion, then, our primary indication "relates to diet and exercise both of mind and body," a strict attention to which, in the slighter cases, with a mild aperient, will often effect a cure. For reasons pointed out before, "to eat moderately and slowly, is often found of greater consequence than any other rule of diet." The first feeling of satiety should, of course, be attended to by the dyspeptic patient; "a single mouthful taken after this, oppresses a weak stomach." Certain kinds of food, however, will generate flatus, or run into fermentation, with every precaution as to quantity. These, which are found out by individual experience, must be guarded against.

"Acescent, and oily articles of food, with a large proportion of liquid, compose the diet most difficult of digestion. It would appear that a feeble gastric fluid, as indeed we might *à priori* suppose, does not admit of being greatly diluted without having its powers much impaired. The diet opposite to this, then, is that which agrees

---

* See our review of Dr. Yeats' paper, in the 4th number of this series, p. 639, *et seq.*

best with dyspeptics. In the first stage of indigestion, a diet composed pretty much of animal food and stale bread, is the best." 121.*

All mucilages being of difficult digestion, and young animals containing more of this than old, the flesh of the latter is to be preferred. Vegetables are still more difficultly acted on by the human stomach, and must be used with caution.

"From what it arises that mutton is to most stomachs so much more easy of digestion than beef it would be difficult to say. Most kinds of game are of easy digestion. Fish, independently of the heavy sauce with which they are eaten, are, for the most part, less easily digested, than the flesh of land animals; and as they at the same time afford less nutriment, they are in both respects less proper for the food of dyspeptics; although from the white kinds being less apt to excite fever, they, like the animal mucilages, have obtained the name of light, a term which so often deceives with respect to what is most easy of digestion, that it is necessary to keep this explanation of it in view."

Of all meats, Dr. Philip considers the lean part of venison as the most digestible. Hare and partridge appear to be as much so as mutton. Pork and tongues, as most mixed with fat, are, *ceteris paribus*, most oppressive; and, of the poultry tribe, geese and ducks are least likely to agree with the dyspeptic stomach. New bread is well known to form a pultaceous mass which is, with difficulty, pervaded by the gastric juice. For many useful remarks on the kinds and the cooking of foods, we must refer to the work itself. We shall make room, however, for the following passage:—

"It is not generally known, that the most consentrated decoction of beef, so far from nourishing, will not, if unmixed with something solid, even allay the appetite. A person under my care was attacked with severe pain of the face when even the smallest quantity of any solid food was put on the stomach, a single mouthful of bread never failed to bring on the attack; and, as he at length refused all solid food, he was confined for some weeks to a strong decoction of beef; but, however strong, and in whatever quantity it was taken, it never relieved the calls of hunger, and he rapidly emaciated." 126.

In respect to liquids, it is certain that, when the various functions are in the due balance of health, but small quantity is necessary—the inhalation by one set of vessels nearly compensating for the exhalation by others. But in indiges-

---

"* The principles in human food," says Merat, "most nutritive and easy of digestion, are the farina of vegetables and gelatin of animals. In proportion to the abundance of these, is their utility in indigestion."—*Dict. des Sciences Med. Tom.* 24.

tion a false thirst is often excited by the irritation of the undigested food in the stomach. An exclusive dogma has, of late years, been set up that no fluids should be used at meals:—this is just as erroneous as the opposite opinion, that we should drink with every mouthful, to dilute the food. The truth appears to be, that in perfect health, and when the skin is not acting beyond the medium range, a person feels little inclination for drink during the time of eating; but there are so few of us in this state of primitive health, that the rule is quite absurd when generally or universally applied. We must drink in moderation when we feel thirst, both at and after eating. We should like to see the advocate of *dry feeding* at dinner beneath a tropical sky! He would soon abandon his hypothesis—or at least the practical application of it.

"The best rules, I believe, which a dyspectic can follow, are not to yield to every slight sensation of thirst, and when the sensation is considerable, to take but a moderate quantity, and that deliberately, for it is with drinking as with eating, if he swallows with too great rapidity, he will take too much." 134.*

---

* Zenophon informs us, that (what he terms) the ancients seldom drank till the repast was finished; and Mr. Dodwell, in his recent travels in Greece, asserts that, to this day, the same custom prevails. "When the dinner is finished, a draught of wine is taken by each person."—Vol. I. p. 156. It is evident, however, that in the time of the Romans, and long before that period, drink was introduced at meals. We are informed by Persio that the Romans drank hot water at dinner as a luxury, and to excite the appetite and digestion.—*Ant. Persio del Bever caldo, &c. Venet.* 1593.

The following sentiments of Messrs. Hallé and Nysten will be found, we imagine, more agreeable to nature and truth than the excentric dogmas of a certain school in this country.

"La quantité de boisson à prendre pendant le repas doit être en proportion d'autant plus grande ou moindre, que les alimens eux-mêmes sont plus secs ou plus humides; qu'ils se laissent plus ou moins aisément pénétrer par les liquides salivaires et gastriques; qu'ils forment, par leur viscosité, une masse plus ou moins tenace; qu'ils ont qlus ou moins la propriété de distendre l'estomac et d'y séjourner un certain temps.

"Les boissons doivent aussi être prises en quantité plus ou moins grande, suivant les constitutions individuelles qui, en raison de leur degré de sécheresse ou d'humidité, présentent des différences très-grandes relativement à la quantité et au degré de liquidité des sucs salivaires et gastriques. Les personnes sèches et bilieuses, dont les organes sont très-irritables et dont la chaleur propre est plus ardente, dont les évacuations intestinales sont plus habituellement dures et sèches, qui sont ordinairement constipées, ont besoin d'une plus grande quantité le liquides aqueux et frais.

"La proportion des boissons aux alimens doit enfin varier selon l'influence des saisons et de l'état de l'atmosphère.

"On peut cependant poser en principe, 1° qu'une quantité de boisson

While our able author agrees with all the best physicians of this and other countries, that water is the best beverage, in a state of health, yet he is forced to acknowledge that, from the long prevalent habits of society in this country, "more or less alcohol is necessary to support the usual vigour of the greater number of people even in health." Nothing therefore could be more injudicious than wholly to deprive them of this support, when they are already weakened by disease." Dr. Philip considers cider as the best fermented liquor of this country, provided the acetous fermentation has not commenced. Perry usually contains too much mucilage—"the home-made wines are still more objectionable," as more disposed to acetous fermentation. The form in which alcohol does least harm is that of foreign wine. "The astringent property of port wine seems to give it a peculiar tonic power; and, if it do not constipate, there is perhaps no other wine so well suited to dyspeptics." Distilled spirits, though they increase digestion for the moment, induce debility by the over-excitement.

"A very moderate use of wine can hardly be said to be injurious; we see those who use it in this way, live as long, and enjoy as good health, as those who wholly abstain from it; and to some constitutions, independently of the effects of habit, it may be useful. I believe neither of these observations apply to distilled spirits, although, as already hinted, when the stomach has been greatly weakened by excess, so that it cannot digest any fermented liquor which has not been distilled; the effects of diluted spirits are often less injurious, than the total collapse of the system which ensues on wholly withdrawing the accustomed stimulus." 145

A moderate use of black tea, and also of coffee, appears to our author to be innocent. Of this we have no doubt. Dr. Philip does not recommend fluids of a very high or very

---

qui excède trop la mesure des besoins naturels, énerve les digestions et favorise les altérations spontanées des alimens qui séjournent dans l'estomac, surtout quand ce viscère a peu d'activité; 2°. qu'une quantité de boisson insuffisante prolonge le séjour des alimens dans la cavité gastrique, et entretient le sentiment de plénitude qui en est la suite. Mais il faut surtout, à cet égard, se mettre en garde contre l'habitude, qui outrepasse plus souvent la mesure qu'elle ne reste en-deçà; connaitre, par son expérience, quelle quantité de liquide est la plus favorable; savoir que la soif que donne l'usage des substances sèches, en épuisant sur-le-champ les organes salivaires, n'est souvent que momentanée et se dissipe en peu d'instans par le renouvellement de la salive. Ces observations sont importantes pour ceux dont les digestions sont lentes, imparfaites; pour ceux qui sont sujets aux aigreurs, et chez qui les fonctions de l'estomac sont aisément troublées par la superfluité des liquides."—*Dict. des Sciences Med. Tom.* 3. *p.* 222.

low temperature. Both extremes he thinks injurious. He is not an advocate for eating little and often. Regular meals at stated intervals appear the best practice.

"It has appeared to me that with the generality of dyspeptics, to take three moderate meals in the twenty-four hours is the best rule. A few find four meals better. The last meal should always be taken a little before bed-time, and should never, particularly after the disease has continued for some time, consist of animal food. The dyspeptic should eat nothing in the intervals of these meals. There is no greater mistake than that he should constantly be taking something. This disturbs the natural process, and entirely prevents the recurrence of appetite, a certain degree of which is a wholesome stimulus to the stomach. The stomach by this constant eating becoming more and more debilitated, and every part by sympathy partaking of the debility, the patient wholly misapprehends the cause, and with a view to increase his strength still increases the frequency of his meals, till he hardly passes a couple of hours without eating. By such a practice, pursued for years, I have repeatedly seen debility of the stomach and a morbid irritability of the whole system established." 151.

On the subjects of exercise, air, and sleep, Dr. Philip makes many useful observations, which, however, are not particularly novel to the medical reader.

In respect to the medicinal remedies in this stage of indigestion, our author "attempts no other division than the simple one of those which act directly on the stomach and bowels, and those which influence them through other parts." To give any curative plan a chance of success, as we before stated, it is necessary not only to remove the causes, and prevent their re-application, but also to remove the more immediate effects of these causes, and thus prepare, as it were, the digestive organs for the operation of the remedial plan of treatment.

As the stomach and bowels are generally overloaded, when we first see a dyspeptic patient, our author considers an emetic, followed by some mild aperient, as usually a necessary preliminary. Of late years emetics have been too much neglected, and there seems a dread of them among patients as well as practitioners. They are certainly rough remedies, but they are very useful ones occasionally. Their repetition should be avoided as much as possible.

"When it appears that offensive matter still remains in the stomach and bowels after the operation of the emetic and aperient, which may be known by a sense of oppression and distention of these cavities, and by eructation of wind and ill-digested food, or of an acid matter, which is sometimes so acrid as almost to excoriate the fauces; we must by gentle stimulants, particularly the distilled waters occasionally mixed with a small proportion of some aromatic tincture, endeavour to excite them to a better secretion; and at the same time by the use of correctives, more directly to alter the morbid properties of their contents." 174.

Acidities in the stomach must, of course, be corrected by alkalis; and although the generation of these acidities may be lessened, *pro tempore*, it cannot be wholly prevented by animal diet. Dr. Philip has repeatedly observed that, on exclusive animal food, the contents of the stomach and the breath become very acid, as soon as the patient begins to feel disgusted with it.

If the pains and spasms arising from the irritating secretions of the stomach and bowels are not relieved by the above means, or aromatic tinctures in addition, then Dr. Philip recommends an opiate, either combined with, or followed by, an aperient, to obviate its constipating effects.

Diarrhœa must be restrained by opiates and absorbents —vomiting by the effervescing draught, "or a mixture of sulphuric acid, conserve of roses, and mint water, carefully strained." When these fail, Dr. Philip has known a pill composed of opium and camphor the most effectual remedy, together with blisters to the region of the stomach. Dr. Philip does not consider pyrosis as necessarily dependent on indigestion.

Having thus brought the stomach and bowels as nearly into a natural state as their debilitated functions will admit of, we are next to consider how their tone may be sustained. The remedies for this purpose are thrown into two classes by our author—those which excite, for the time, the particular functions of the digestive apparatus, or allay the nervous irritation thereof—and those which bestow some degree of permanent vigour. The first class comprehends stimulants and anodynes—the second, bitters, astringents, and eccoprotics. A combination, indeed, of aromatics with bitters and aperients, will generally answer better in these cases, than any class exhibited singly. Among the useful stimulants, Dr. Philip places ammonia and its carbonate, which, he thinks, have not perhaps obtained all the attention they deserve in this disease.

"They are more apt to heat than aromatics, and, in the same proportion, more beneficial in that languor and coldness, which are often such prominent features of indigestion. Their greater tendency to heat seems to arise from their acting as a more general stimulus. They are more apt to strengthen and quicken the pulse, and, probably, act on the sanguiferous system after they are received by absorption;

I have found them decidedly serviceable when aromatics had failed. They are best adapted to those cases where a continuance of the disease has produced much debility, and consequent languid circulation, without much tenderness of the epigastrium, or hard pulse, or any sensation of burning in the hands or feet at night." 180.

External heat to the region of the stomach, and frequently renewed, is often of more service in relieving gastrodynia than internal heat. Warm water internally, however, is no bad remedy, when not abused by too frequent repetition. Very small proportions of opium, as two or three minims of the tincture, two or three times a day, "often prove highly serviceable in allaying morbid irritation," their constipating effects being easily counteracted. We have found pills composed of two grains of extract. col. comp. one grain of pil. hyd. one grain of cayenne pepper, or some oil of cassia, with a quarter of a grain of opium, taken twice a day, or two or three of them at bed time, prove exceedingly useful in the class of complaints now under consideration. Dr. Philip prefers the pulvis ipecacuan. comp. from two to four grains given every six or eight hours, discontinued and renewed from time to time. For what are termed, and perhaps not improperly, *nervous* symptoms, castor, myrrh, valerian, and assafœtida, afford temporary relief—especially to that palpitation which results from indigestion, provided no inflammatory disposition has supervened. But for the prevention of a recurrence of indigestion, "bitters and astringents are those on which we chiefly rely," their effects being increased by combining them with a small quantity of the stimulant before alluded to. Of the bitters, chamomile, orange peel, and wormwood, appear to be most devoid of stimulating principles. Calumba possesses this quality in a greater degree than gentian and cascarilla—cinchona most of all.

"All the foregoing bitters, if we except the bark, which is often oppressive to the stomach, are well suited to the first stage of indigestion; but in proportion as the second stage approaches, we find the less stimulating bitters answer better; and in the second stage, even the gentian, which, of those that deserve the name of stimulating, possesses, perhaps, the least of this property, is often too heating, and the bark in general cannot be borne, even for a few days. While in the earliest periods of the disease, when it supervenes on debilitated states of the constitution, and the stomach still retains considerable comparative vigour, a cold infusion of the bark is often the most beneficial of all bitters." 185.

Of astringents, which Dr. Philip thinks, must sometimes be resorted to, at the expense of correcting their effect on the bowels, iron deserves the first place. "In chlorotic indigestion, combined with stimulants, it is the most powerful medicine we possess;" and there are, Dr. P. thinks, "few cases of indigestion in which it is not found more or less useful at an early period." Its good effects are increased by combining it with bitters and aromatics. Next to this, Dr. Philip considers the sulphuric acid as the best stomachic astringent, especially where sweating is too easily induced by exercise. The white oxyd of bismuth has lately been much commended in gastrodynia. Sarsaparilla is much esteemed by our author, though not so much in early stages, as in protracted cases. In the early stage, and while the disease is confined to the alimentary canal, mercury he conceives to be unnecessary, if not prejudicial.

Aperients, of course, hold a distinguished place in all stages of the disease. Our author has found none, employed merely for the purpose of supporting a regular action of the bowels, so generally useful as pills composed of ipecacuanha, compound extract of colocynth, and soap, taken occasionally at bed time.

The stomach and alimentary canal may also be acted on sympathetically through the medium of the skin. Tepid and cold bathing, friction, blistering the epigastric region, and covering the same with stimulating and anodyne plasters, are useful auxiliaries, in the treatment of indigestion.

When the alvine discharge begins to deviate from the healthy state, the treatment becomes more complicated. The secreting power of the liver, and probably of the pancreas, participates in the derangement. We must now, therefore, combine with the foregoing remedies, such others as tend to "correct the morbid state of the liver." Mercury is our principal remedy in this case, and the great art consists in so managing its exhibition, that it shall produce as little injury as possible to the other parts of the system.

In the first stage of indigestion, there is no necessity that the remedy should enter the system. Its local effects on the primæ viæ and liver are sufficient. Neither should the medicine be long continued, even in this restricted manner, without attending to the state of the alvine discharge, and ascertaining the necessity for its continuance.

"In the more obstinate cases indeed where the disordered state of the liver constantly recurs at short intervals, it is better for a certain time to give a moderate dose at stated intervals, by which the alimentary canal will suffer less, as a smaller dose is required for the prevention of this state, than for its removal." 199.

In this early stage of indigestion, inunction is not necessary. Calomel and blue pill are the two forms in general use—the former, most aperient, should be given when the bowels are most languid—the latter, when they are more easily excited. "The blue pill is generally most oppressive to the stomach; the calomel most irritating to the bowels." But as small doses often hang in the bowels, so they are often more irritating than larger doses which more quickly carry themselves off. "The irritation of the bowels is most effectually prevented by taking an opening draught some hours after the calomel." Dr. Philip has known a warm mercurial plaster worn over the region of the liver, for months and even years, with great advantage, in cases rather obstinate than severe, "the symptoms constantly recurring when it was laid aside." The mineral acids, and in some cases, dandelion Dr. P. considers the best substitutes for mercury. But they seldom maintain a due action in the liver for any length of time. Finally, Dr. P. sums up thus:—

"The treatment of the first stage of indigestion, then, consists in promoting the due action of the stomach and bowels, by the various means which have been detailed, and correcting the secretion of the liver, if it deviates from the healthy state, by the occasional use of mercury; care being taken neither to employ it in greater quantity, nor for a longer time, than is necessary for this purpose, as its effects on the stomach and bowels are evidently in opposition to the other parts of the treatment." 209.

*Treatment of the Second Stage.* This stage generally does not take place till the function of the liver "has been disordered for some time, or its disordered state has repeatedly occurred." When this stage is established, "bitters and aromatics cease to give any effectual relief"—indeed, in many cases, they increase the feverish restlessness. More tonic medicines have a still worse effect.

"The patient often thinks that his disease admits of no relief, but from aperients, and particularly mercurial aperients, of the good effects of which he is always sensible, and, consequently, is very apt to fall into an excessive use of them." 211.

Dr. Philip considers that we should be guarded in the use of purgatives and mercurials, since, in the second stage, the epigastric tenderness and hard pulse leave little doubt that inflammatory action, or a state approaching to it, has there supervened. "Stimulating measures are therefore to be employed with more caution, and anti-inflammatory measures become more or less necessary." The application of leeches to the tender part of the epigastrium appeared to our author to throw light upon the nature and treatment of the second stage of indigestion.

"The effects I found were not merely that the tenderness was relieved, and the pulse softened; but that the patient breathed and walked better, that the bowels were more easily moved, and the skin appeared more relaxed, the feverish tendency which frequently shews itself in the evening, being in the same degree lessened." 217.

But this was not all. On resuming the plan of treatment, it soon appeared that the patient bore the use of tonics much better than before, and, in some instances, a recurrence to the plan of treatment pursued in the first stage removed the disease. But such fortunate cases were comparatively few, "and a repetition of the leeches became necessary."

"Each repetition to the same extent generally produced less relief than the preceding, and if a larger quantity of blood was taken, the relief was obtained at too great an expense of strength.

"The application of a blister to the part from which the blood was taken, immediately after its abstraction, I found, tended both to increase the effect of the leeches and render it more permanent; but even with this aid their repetition in the more inflammatory cases soon became necessary. In those less inflammatory, blisters sometimes relieved the symptoms without the aid of leeches, but like the leeches they often failed to give permanent relief." 219.

The lighter bitters, as chamomile or orange peel, and even occasional aromatics, can still be borne, and a very little light animal food, as chicken once in two days, supports the strength better than a diet composed wholly of animal food. Where wine is given at all, it should be well diluted. If active inflammation be threatened, the diet must of course be reduced to the lowest ebb.

"In a few, particularly when a considerable degree of hardness of pulse, notwithstanding the use of the above means, continued, I have seen a diet wholly vegetable, and even a total abstinence from wine, which is much less permanently stimulating than animal food, strikingly beneficial. It is common for the appetite to improve on lessening the quantity of animal food. This depends in part on other food affording a less proportion of nourishment, but very much, I believe, on the tendency to fever being lessened by the change." 221.

The bowels are often not only torpid under the use of animal food, "but purgatives act imperfectly, and with great irritation." Vegetable diet frequently relaxes them, without the aid of medicine.

To obviate the inflammatory tendency in these cases, Dr.

Philip has found nothing equal to a very dilute solution of nitrate of potash, with a little gum. The latter seems to defend the bowels from the irritation of their contents.

"Eight or ten grains of the nitrate in an ounce and a half of water with a twelfth or sixteenth part of mucilage of acacia, have been given three times a day, and repeated every hour or hour and half, when the skin became hot generally, or the hands and feet began to burn. Two or three doses thus taken seldom fail to reduce the increased temperature, and relieve the restlessness which it occasions; and thus simple as those means are, they often procure good nights, when the want of sleep, as frequently happens in this stage of the disease, is the effect of feverishness. The common saline draught, the sulphate of potash and other medicines of this description have similar effects, but none of them appear to me equal to the above nitrate." 223.

In addition to these means a freer use of aperients will be necessary. Acrid or drastic purgatives are manifestly improper. If the inflammatory symptoms still continue to recur, Dr. P. recommends a perpetual drain, "established in the most tender part." As greater or less disorder of the liver is "a constant attendant on the second stage," "we still find mercury by far the most efficacious." 225. In the earlier periods and milder forms of the *second* stage, the *local* effects of mercury, especially of the blue pill, kept up for some time, will often be sufficient, provided the antiphlogistic measures have been duly employed. "But in many cases, and in a large proportion of those of long standing, this effect often fails." Our author's plan then was as follows:—

"I have generally given a grain of the blue pill, sometimes only half a grain, twice or three times in twenty-four hours, till the secretion of bile appeared to be healthy, repeating these doses when it was again disordered; and by such doses, which may appear to many little better than trifling, I have seen the bile gradually restored to a healthy state, when larger doses had been employed in vain. They not only often succeed where larger doses fail, but the change, in proportion as it takes place more slowly, seems generally to be more permanent." 228.

That the above plan, which a good deal assimilates with that of Dr. Ayre, of Hull, may prove equally efficacious in the hands of others, we sincerely wish. In our own practice, which has not been very confined in these complaints, we did not trust to such cautious administrations of the remedy, and therefore we cannot offer an opinion on Dr. Philip's plan. We present it for the trial of our brethren. The doses of the medicine were so small that, to use the author's own words—"if they did little good, nothing, at least, was to be apprehended from them:"—unless the loss of time, which, by the way, is sometimes of great consequences in such cases.

We can easily conceive, with our author, that—

"The beneficial effects of this plan appearing slowly at first, discourages the hopes of the physician, and, as I know, in many instances, has caused it to be laid aside." 230.

Dr. Philip avers, however, that twenty years' experience has convinced him of the utility of the measure. Diet, exercise, occasional cathartics, local blood-letting, blisters, and cooling medicines, must occasionally lend their aid to the foregoing process. When the blue pill irritates the bowels, extract of poppy, hyoscyamus, or conium, must be combined with it.

It is to the second stage that the substitutes for mercury are best adapted, particularly the nitric and hydrochloric acids, and the taraxacum.

"Much has of late been said of the external use of these acids. Both their internal and external use has appeared to me best adapted to cases of some continuance, where the inflammatory tendency has been to a great degree subdued, and small doses of mercury have been employed without the usual benefit, In such cases, I believe, the use of the acids will almost always be found better than increasing the quantity of mercury beyond what produces the slightest indication of its presence in the gums. If the habit bear the mercury well, the acid may be used in aid of it; if not, or if the use of the acid, as sometimes happens, causes the mercury to irritate the bowels, the latter should be discontinued under the use of the acid." 235.

A little farther on Dr. Philip makes this observation:—"According to my experience, the external use of the acids, recommended by Dr. Scott, is much more powerful, both as a substitute for mercury, and a means of correcting its debilitating effects, than their internal use." It therefore requires more caution where an inflammatory tendency is suspected. The taraxacum appears to our author, and indeed we can verify his statement, "to possess greater powers in this disease, than are usually ascribed to it; but it requires to be taken in very large doses." Sarsaparilla is also no mean auxiliary, particularly where much debility attends, and on the same principle, "a clear and fresh air is often of the greatest use." Our author has seen decided good effects, "when the pulse was much contracted, and the skin shrunk and cold," from very small doses of the colchicum; but it must be used with caution. Our author makes some judicious observations on change of air, and exercise of body, for which we must refer to the volume itself.

On the sympathetic affections occurring in other organs than the stomach, during indigestion, Dr. Philip makes also many interesting remarks, some of which we shall notice in a cursory manner. The nature of these remote affections corresponds with that of the stomach itself. In the first stage they are merely nervous—ceasing with the cause that produced them. In the second stage they are inflammatory, having an existence independent of that cause. These secondary symptoms of indigestion are most common in youth, and least so in advanced periods of life; *i. e.* after forty. The liver is generally the first organ to suffer, both in function and structure. It is not uncommon, therefore, in the second stage of indigestion, for the right hypochondrium to swell and become tender on pressure, with a sense of oppression and increased hardness of the pulse, often accompanied with some dyspnœa and dry teasing cough, together with a deranged state of the biliary secretion—" in short, the patient evidently labours under inflammation of the liver." For these attacks local bleeding, blisters, saline aperients, and the blue pill, are generally sufficient. Dr. Philip has seen the spleen occasionally affected as the disorder of the liver gave way, and vice versa.

The lower bowels are often sympathetically affected—partly from their consent with the stomach, and partly perhaps from the irritation of disordered secretions passing through them. In the first stage of the disease purgatives and anodynes are generally sufficient; but in the second stage there is an inflammatory tendency, characterized by fulness and tenderness in the hypogastrium.

" The sigmoid flexure of the colon appears to be the part most liable to be affected, probably from the contents lodging there longer than in other parts of the large intestines. It is not uncommon in protracted cases, to find a considerable degree of tenderness in the seat of this part, which is sometimes at length affected with ulceration. It is, also, probably for similar reasons, common, though not so much so, to find the tenderness on pressure in the seat of the cœcum." 261.

The application of leeches, and the use of mucilaginous and anodyne glysters, are most proper here; "and then mild aperients generally succeed in procuring a free action of the bowels."

The chest frequently suffers in the second stage of indigestion, but still oftener the head. The heart also is frequently affected sympathetically, and palpitation and other alarming symptoms produced.

"It is a common observation that carditis is apt to supervene after repeated attacks of rheumatic pains of the limbs. I believe from many cases which have fallen under my observation, that it will generally be found in such instances, that the rheumatic pains had been combined with, and in a greater or less degree dependant on, disorder of the digestive organs." 265.

Dr. Philip having now traced indigestion from its commencement to the moment at which it is about to terminate in organic disease, declines entering into this wide field, since to give any thing like a regular account of the structural changes in various organs resulting from long-continued disorder of the digestive apparatus, would require a larger space than the whole volume. He therefore singles out dyspeptic phthisis, a disease of which he formerly published an account in the Medico-Chirurgical Transactions, for the remainder of his treatise; but as it is only an amplification of the paper so universally known to the profession, we shall decline entering on it here.

Our readers must have remarked that the etiology, pathology, and treatment of the important class of diseases treated of in this volume, and of which we hope we have presented a fair analysis, are founded on *observation and experience*,* and not on any hypothesis connected with the experiments which Dr. Philip has made on the digestion of animals. Had his experiments, therefore, been negatived by the trials of others, such failure would not, in the slightest degree, have affected the present volume. We are, however, in justice bound to state, that the experiments alluded to, and which have lately excited so much attention and discussion in the medical world, were recently repeated at the Royal Institution, in the presence of Sir Humphry Davy, Mr. Knight, Mr. Brodie, Mr. Broughton, Dr. Darling, and many other medical gentlemen, among whom was the editor of this Journal; and that Dr. Philips's statements were fully, unequivocally, and satisfactorily verified, without leaving the smallest doubt of their correctness on the mind of any one present. Mr. Brodie and Mr. Broughton, with that candour and liberality characteristic of great and philosophic minds, gave every assistance to Dr. Philip, and when the results were developed, most handsomely and ingenuously avowed their conviction of the truth of his former statements, and consequently the verification of his experiments.

---

* Some few parts of the pathology of indigestion are founded on *facts* ascertained by opening animals at various periods after eating, and therefore are not to be deemed hypothetical.—*Rev.*

As some curious phenomena became apparent during the performance of these operations at the Royal Institution, we shall here present a very succinct, but we hope intelligible, view of the state of the question, derived almost exclusively from what we ourselves observed.

As a preliminary we may remark that the stomach of a rabbit does not appear capable of completely emptying itself, however long the animal may fast; but on the introduction of new food, the latter occupies the centre, and the former the inner circumference of the muscular pouch. The old, or digested food, is then forwarded along the great curvature of the stomach to the pyloric orifice, while a layer of new food comes in contact with the organ—becomes digested in its turn—the layer or stratum is forwarded in the same manner as the other, and so on, layer after layer, till the stomach disgorges into the duodenum all that it is capable of sending there.

Now if three rabbits be kept sixteen or seventeen hours without food, then fed with green parsley, and the eighth pairs of nerves, in two of them, divided, in the neck, immediately after feeding, we have three subjects for experiment. In that rabbit which is *not* to be galvanized, a piece of each nerve must be cut out, for reasons which will presently appear. The inferior cut extremities of the nerves in the other animal are to be freed for a small space, so as that a piece of tinfoil may be tied on each, and the two pieces of tinfoil joined together, so as to form a common conducting medium for the galvanism. The positive wire is to be kept in contact with this union of tinfoil, and the other wire kept in contact with a piece of silver at the pit of the animal's stomach. The wires are to be so managed as to keep such a stream of galvanism passing along the eighth pair as may cause a constant twitching of the fore legs. We shall soon perceive that the rabbit, with the nerves divided and allowed to run about, begins to breathe with difficulty, and with noise. The animal on the table, under the same circumstances, but galvanized, breathes easy, and makes no noise, provided the laryngeal nerves are not so injured as to occasion a straightening of the glottis. In two or three hours after the division of the nerves, the non-galvanized rabbit makes efforts to vomit:—the galvanized animal makes no such efforts. If both animals live six, seven, or more hours, and are then killed, together with the third rabbit, which has undergone no operation, the food in the healthy rabbit will be found digested, and uniform in its appearance, and greatly diminished in quantity; nearly, if not exactly, the same appearances will present themselves in the galvanized rabbit; while in the non-galvanized rabbit, the food will present one of two appearances, according to the length of time the rabit lived after the nerves were divided. If the animal survive but a few hours, as four, six, or seven, a layer of the old food will be seen on the surface of the new,—the latter being quite undigested. If the rabbit survive fifteen, twenty hours, or more, the stomach will have got rid of its old food, and the whole of the new undigested food will be in contact with the stomach at all points; but there being no nervous or galvanic fluids to excite the secretion of gastric solvent, the parsley will be every where and uniformly undigested, and nearly in the same state, as when first masticated—at least it will merely be a little sodden, from the heat and moisture of the place where it had lain so long. Such being the state of things, the natural, and apparently inevitable, inference is, that the galvanic fluid, in the galvanized rabbit, supplies the place of the nervous influence, and furnishes the *exciting cause* of digestion in the stomach.

We are now to notice a circumstance which, we believe, gave origin to all the discussions and discrepancies of opinion which have appeared respecting this curious physiological question:—it is this. If the nerves of the animal are simply divided, but no piece cut out, or other means taken to prevent the divided ends coming into contact, or proximity, then an irregular but imperfect digestion takes place, and the experiment leads to no positive conclusion. This was the case in the first train of experiments made at the Royal Institution, and we believe this was the case also in the experiments of Messrs. Brodie and Broughton. From this it would appear, that while the cut extremities of the nerves are in contact or proximity, the nervous influence is transmitted, though imperfectly, along the original course of the par vagum, from the brain to the stomach, and a corresponding degree of digestion is carried on—the animal dying apparently from hydrothorax, with lungs perfectly collapsed; whereas, when the divided ends of the nerves are kept separate, there is no hydrothorax; but the lungs are so congested, as to retain the form of the chest after it is laid open. In the galvanized rabbit, on the other hand, provided the galvanism has not been long enough continued to produce inflammation, there is neither congestion nor hydrothorax, nor the patching on the surface of the lungs, which appears in both the former cases—but the lungs have a perfectly healthy appearance.

In one respect, Dr. Philip's opinions have been more or less misconceived by, we believe, all who have publicly noticed them; although he has been at great pains both in his inquiry into the laws of the vital function, and in several numbers of the Journal of the Royal Institution, to explain this part of them.

In the former of these publications he first stated the facts from which the identity of the nervous influence and galvanism appeared a necessary inference. But his definition of the term nervous influence has been wholly overlooked in commenting on his opinions. Le Gallois is the first writer, as far as we know, who has attempted experimentally to distinguish between the sensorial and nervous powers. It appears to Dr. Philip, however, that the line of distinction which Le Gallois attempts to draw between these powers is incorrect, and he endeavours, by observing the process of dying, to draw one more consistent with the phenomena.

It appears from many experiments and observations detailed in his "Inquiry," that after the sensorial functions cease, where the animal is no longer capable of perception or any act of volition, and consequently is said to be dead, the nervous as well as the muscular powers still survive; although the former, from the ceasing of respiration, to which he has shewn that the sensorial power is necessary, is greatly impaired. He has shewn by repeated experiments that the nervous power is still capable of forming the secreted fluids, and of causing an evolution of caloric from the blood, as well as of exciting the muscles. He thus determines not only that the nervous power is distinct from the sensorial, but capable of its functions after the latter is withdrawn.

Those who have commented on his opinions have not only confounded what he calls the nervous with the sensorial power, but with the vital principle. In the 15th number of the Journal of the Royal Institution, he observes,

"Were it not for opinions, unaccountably ascribed to me, I should think it superfluous to add, that I have never regarded galvanism as having any thing in common with the sensorial and vital powers, as I have explained in the first, and still more fully in the second edition of my *Inquiry*. I only maintained that we have reason to believe that the nervous influence which I had, with much pains, and the assistance of many experiments, attempted to define; shewing, that it survives the sensorial power, but is wholly incapable of its functions after the of extinction the vital principle, is a chemical agent: and that it is the same which operates in many similar phenomena which we know to be the effects of galvanism."

Dr. Philip conceives that his experiments prove, that the nervous influence, properly so called, performs no functions but such as are evidently the effects of a chemical agent, and, as he arrives at this conclusion before he begins to enquire into the nature of the chemical agent employed by nature in the production of the functions in question, it is incumbent on those who dispute his opinion, in the first place, to enquire how far he is successful in establishing this previous inference. If he be correct in thus limiting the operation of the nervous influence, and can prove that galvanism is capable of the functions to which he thus limits the nervous influence, the identity of these powers, as far as we are capable of judging, can no longer be doubted.

Besides, we have seen, in the experiments made at the Royal Institution, that the nervous influence is capable of passing along a nerve after its division. Now, after the division of a nerve, more or less moisture must intervene between its divided ends, even if every pains be taken to prevent it by adapting, as correctly as possible, these ends to each other; but, in the above experiments, no pains of this kind were taken; the divided ends of the nerves were merely allowed to fall into their place. Here then it is proved, that the nervous influence passed through water. This proves that it does not depend on a vital action of the nerve, and, we need not add, goes far of itself to establish the position of Dr. Philip respecting its nature. We here see it passing through a conductor of galvanism.

The readers of this Journal will have perceived that, in the foregoing article, as indeed in most others, we have pursued a strictly analytical path, and stated, in the last few pages, what we believe to be truth—and an impartial view of the physiological question, now, we hope, set at rest. Of the general merits of the work, we leave the public to form their own opinion, having set before them sufficient matter whereon to ground their judgment.

